# A Rare Case Report of Giant Urinary Bladder Stone Causing Recurrent Dysuria in a Woman

**DOI:** 10.1155/2022/4835945

**Published:** 2022-08-27

**Authors:** Rojan Adhikari, Hari Prasad Baral, Utsav Bhattarai, Ravi Kiran Gautam, Kiran Jung Kunwar, Dipesh Shrestha, Bijay Mansingh Katwal

**Affiliations:** Department of Urology and Kidney Transplant, Shahid Dharmabhakta National Transplant Centre, Bhaktapur, Nepal

## Abstract

**Background:**

Large urinary bladder stones are not common and even less common in females. We report a case of large bladder stone presented with acute retention of urine in a female patient. *Case Report*. A 62-year-old female presented in emergency department with retention of urine for 12 hours with history of recurrent UTIs for last 1 year. She was also complaining of mild dull lower abdominal pain for last 6 months. She had no history of incontinence of urine and fever. On physical examination, hard mass was palpable on suprapubic region on palpation of abdomen. Urine culture shows Escherichia coli for which antibiotics was given. An X-ray kidney ureter bladder showed a radio-opacity in the pelvic region measuring 9 × 8 cm in size. Ultrasonography revealed bilateral mild hydronephrosis with a large bladder stone. Open cystolithotomy was performed, and the stone was taken out. Stone biochemical analysis showed predominantly urate crystals. Patient had uneventful postoperative course, and she was discharged on 4^th^ postoperative day and was followed up for 1 months after operation.

**Conclusions:**

Large urinary bladder stones are not common and even less common in females. Clinician should have think regarding large bladder stone as a cause of recurrent lower urinary tract symptoms like dysuria and should assess renal function for proper treatment. Open cystolithotomy is choice of operation in large bladder stone.

## 1. Introduction

Bladder stones (BS) comprised of 5% of all urinary tract stones. The aetiology of bladder stones is multifactorial. Bladder stones can be classified as primary, secondary, or migratory. Primary calculus are stones which passes from kidney or ureter and lodges in the urinary bladder while secondary calculus are formed inside the bladder [1]. Primary stones occur in the absence of other urinary tract pathology, whereas secondary bladder stones occur in the presence of other urinary tract abnormalities, such as bladder outlet obstruction (BOO), neurogenic bladder, chronic urinary tract infection, foreign bodies, and bladder diverticula. In male, the bladder stones are usually related with urinary obstruction, urinary retention, urinary tract infections, foreign body retention, and enlargement of prostate. While in female, it can occur secondary to bladder outlet obstruction, neurogenic voiding dysfunction, urinary tract infections (UTIs), or foreign bodies. The prevalence of bladder stone is very high in male in comparison to female population. Nearly, about 5% of all bladder stone occur in women. Giant urinary bladder calculi are defined as stones with weight more than 100 g and dimensions larger than 4 cm in diameter [2] . Giant or massive bladder stone is an uncommon condition which usually take long time to form. The presenting symptoms and signs varies with dysuria, lower urinary tract symptoms (LUTS), hematuria, pyuria, and recurrent urinary tract infections. Large bladder stones are common in developing countries because the initial symptoms of lower urinary tract symptoms are usually overlooked [3].

Our patient who is from rural part of Nepal experienced recurrent dysuria without any additional workup and received empirical treatment despite of recurrent symptoms. Here, we report an unusual case of giant urinary bladder stone causing recurrent dysuria in a female patient.

## 2. Case Report

A 62-year-old female presented as an emergency patient with retention of urine for 12 hours. She had history of hesitancy at urination, increase frequency, interruption of urinary stream, urgency, and feeling of incomplete voiding for last 1 year. She was complaining of mild dull lower abdominal pain for last 6 months, intermittent in nature, no radiation, and aggravated while micturition. She had burning micturition on and off. She had no history of incontinence of urine and fever. She smoked cigarette 20 pack per year and left 10 years back. She denied any history of significant weight loss and hematuria. She is hypertensive under medication for 5 years and has chronic pulmonary obstructive disease for 2 years. On physical examination, her vital signs were stable. Hard mass was palpable on suprapubic region on palpation of abdomen. However, there was no renal angle tenderness present, and digital rectal examination was not done. Her blood investigation showed normal white blood cell count, blood urea nitrogen, and serum creatinine. Urine analysis indicated a very high white blood cell count and few red blood cells. Urine culture shows Escherichia coli for which antibiotics ciprofloxacin was given.

**Figure 1:**
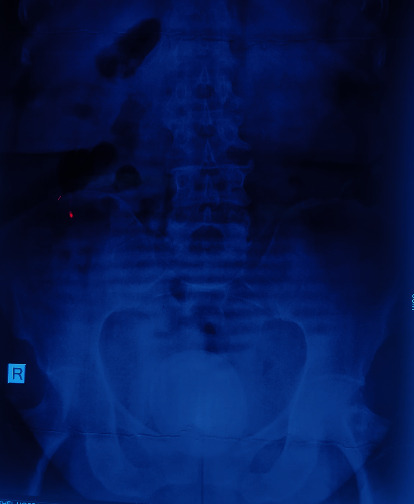
X-ray pelvis with bladder stone.

**Figure 2:**
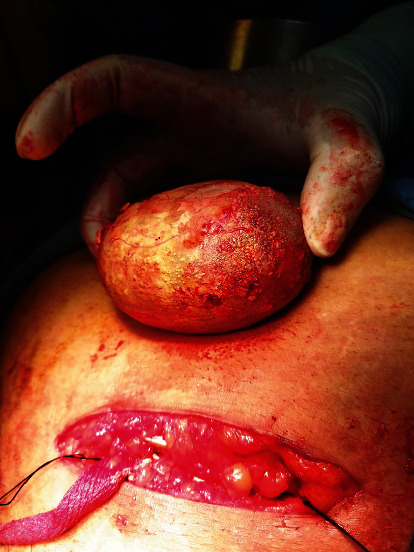
Stone after removal from bladder.

**Figure 3:**
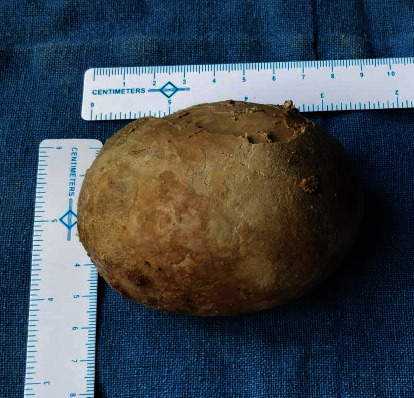
Size of bladder stone.

An X-ray kidney ureter bladder showed a radio-opacity in the pelvic region measuring 9 × 8 cm in size (Figure 1). Ultrasonography revealed bilateral mild hydronephrosis with a large bladder stone. After aseptic urine, cystoscopy was performed which shows no any lower urinary tract obstruction and large bladder stone almost occupying the bladder. No additional stones were noticed in the urinary tract. After optimization, open cystolithotomy was performed under spinal using a suprapubic, midline incision. A transverse incision was given in the bladder, and the stone was taken out (Figure 2). The bladder mucosa was erythematous and edematous, with no evidence of masses. The ureteral orifices were visible with normal pouring of urine. Bladder mucosa biopsy was not taken. The bladder was closed in two layers, and a suprapubic catheter of 12Fr and urethral Foley catheter of 16Fr were kept. A retrieved stone was of size 9 × 6 × 7 cm (Figure 3) and a weight of 300 gm. Stone biochemical analysis showed predominantly urate crystals.

After optimization, open cystolithotomy was performed under spinal anesthesia. Patient had uneventful postoperative course, and she was discharged on 4^th^ postoperative day with suprapubic catheter and urethral catheter in situ. Suprapubic catheter was removed on 10^th^ postoperative day, and urethral catheter was removed on 14th POD in outpatient department. She was followed up for 1 month after operation and no any residual stone seen and patient had normal urination.

## 3. Discussion

Bladder stones are commonly contained with mixed composition. Struvite (ammonium magnesium phosphate) stone is the most common stone in presence of history of recurrent urinary tract infections with urea-splitting organisms such as Proteus, Klebsiella, Serratia, and Enterobacter species that produce alkaline urine [4]. While calcium oxalate is most common without history of UTIs. Nearly, 50% of bladder stones are composed of ammonium urate [5]. They are normally related with a positive urinalysis for nitrite, leukocyte esterase, and blood.

X-ray, ultrasound, computed tomography (CT) scan, or cystoscopy are common methods to make diagnosis of bladder stone [3].

Nowadays, it is fact that open approach is being replaced by newer techniques aimed to be less invasive [6]. There is a broad spectrum of modalities for treatment of bladder stones like open cystolithotomy, transurethral cystolithotripsy, shock wave lithotripsy, and percutaneous cystolithotripsy [7]. For our patient, we choose open cystolithotomy as the size of stone is extremely large and choosing other option may lead to retain of stone fragment as described in the literature [8]. Fortunately, the bladder stone was not adherent to the bladder wall, and we can easily take out. We present this case as there is no any bladder outlet obstruction, bilateral ureteric orifice compromised with bladder stone causing mild hydroureteronephrosis, and the bladder stone in female is rare.

## 4. Conclusions

Large urinary bladder stones are not common and even less common in females. Clinician should have think regarding large bladder stone as a cause of recurrent lower urinary tract symptoms like dysuria and should assess renal function for proper treatment. Open cystolithotomy is choice of operation in large bladder stone.
